# Less Than One-Third of Caretakers Sought Formal Health Care Facilities for Common Childhood Illnesses in Ethiopia: Evidence from the 2011 Ethiopian Demographic Health Survey

**DOI:** 10.1155/2015/516532

**Published:** 2015-07-26

**Authors:** Achamyelesh Gebretsadik, Alemayehu Worku, Yemane Berhane

**Affiliations:** ^1^School of Public and Environmental Health, Hawassa University, P.O. Box 46, Hawassa, Ethiopia; ^2^School of Public Health, Addis Ababa University, Addis Ababa, Ethiopia; ^3^Addis Continental Institute of Public Health, Addis Ababa, Ethiopia

## Abstract

*Background.* Most of the childhood illnesses can be proven with effective interventions. However, countless children die needlessly in developing countries due to the failure of their guardians to seek care timely. The aim of this study was to assess health care seeking behavior of caretakers of children under the age of five years for treatment of common childhood illnesses. *Methods.* Further analysis of the Ethiopian 2011 demographic and health survey was done. All children under the age of five reported to have been ill from the three common childhood illnesses and their caretakers were included in the analysis. A complex sample logistic regression model was employed to determine factors associated with the health care seeking behavior of caretakers. *Result.* A total of 2,842 caregivers who reported that their index child had at least one of the three common childhood illnesses in the two weeks preceding the survey were captured, of which 849 (29.87%; 95% CI: 28, 32%) sought formal health care facilities. *Conclusion and Recommendation.* In Ethiopia health care seeking behavior of caretakers for common childhood illnesses is low. Increasing mass media exposure can possibly improve the health seeking behavior of caretakers.

## 1. Introduction

Majority of children in developing countries are dying because of preventable infectious disease. Pneumonia, diarrhea, and malaria remained the major killers of children under the age of five. These three diseases account for more than half of all the deaths in that age group. This is because children are not getting access to appropriate treatment [1, 2].

Prompt diagnosis and appropriate treatment of common childhood illnesses can prevent death and long term illness. However, evidence from several studies show that nearly one-half of caretakers did not take any action within one to two days of their child falling ill [3–6]. According to evidence from many African countries such as Nigeria, Burkina Faso, and Zambia more than half of the children with common childhood illnesses did not seek any medical advice. Others also gave drugs at home or went to a traditional healer/drug shops and only a few of them went to private or government clinics [3, 7]. In many African countries, the median public health facility use for the treatment of common childhood illness in under-five children is below 50% [8].

In Ethiopia, the medical care seeking behavior for the treatment of common childhood illness in health facilities varies from region to region. It ranges from <5% to 72% [9–11]. Among those who sought advice, nearly 73% of care givers sought treatment in government health facilities [9, 10].

In Ethiopia, government is the main health service provider especially in rural areas. Ethiopia is improving the quality of primary health care facilities and its distribution by expanding physical health infrastructure and by providing training and deploying health extension workers to provide basic curative and preventive health care services in every rural community. However, there were many challenges faced by the program due to lack of transportation, bad road conditions, shortage of trained health workers, and unreliable medical supplies, to mention some [12, 13]. In addition, customer handling was reported to be poor to attract caretakers to utilize the service, thus resulting in client and patient dissatisfaction [14, 15].

Maternal education, socioeconomic status of the household, number of children under five years of age in the household, and area of residence were some of the factors related to the utilization of different types of health facilities [4]. Generally, wealthier households are more likely to seek treatment than the poorest; poor households tend to seek care from public facilities, while wealthier households seek care from private facilities [7, 16].

Although provision of treatment for common childhood illnesses in Ethiopia is among the health agenda priorities, few studies have assessed the factors associated with care seeking behavior of caretakers for treatment of common childhood illnesses. Therefore, we undertook further analysis of the EDHS 2011 data for assessing health care seeking behavior of caretakers for common childhood illness using nationally representative data.

## 2. Methods and Materials

The source of the data for the current study was the Ethiopian Demographic and Health Survey which was conducted in 2011 (EDHS 2011). The survey was a large community based cross-sectional survey.

The samples were selected using a two-stage stratified cluster sampling technique. Initially all nine regions were stratified into urban and rural clusters. From 624 enumeration areas (EAs), 187 urban and 437 rural clusters were considered. A total of 18,720 households were included in the survey, 5,610 from urban and 13,110 from rural areas. All care givers with children aged 0–59 months were included in the current analysis.

The children's record data and household members' record data were merged to obtain a working dataset. The dataset provided potential household and individual level predictor variables. The analysis in this study was done in three stages. Firstly, children with at least one common childhood illness (diarrhea, ARI, and/or fever) two weeks preceding the survey were identified and subset of the EDHS data was extracted. Accordingly, dataset with 2,842 records was prepared. Secondly, summary measures were analyzed to investigate caretakers' health seeking behavior for common childhood illnesses. Then, at the third stage, bivariate analysis was conducted using the Pearson's chi-square test to test whether there were a difference between the child, household, environmental, and parental characteristics and treatment seeking behavior of the caretakers. Multivariable analysis using a complex sample logistic regression model was employed to determine factors associated with the health seeking behavior of caretakers for their children under the age of five with common childhood illnesses. SVY syntax was used to weight the analysis. The analysis was made using STATA 12. First, strata were generated using region and residence as stratification variables. Secondly, a normalized weight was generated. Data were declared as complex survey data. A *p* value < 0.25 was taken to select variables for the multivariable analysis. The adequacy of the models was checked using an *F* test.

Some of the variables used for the analysis of this paper were recategorized. A wealth index was provided in the dataset. It was constructed by combining information on household assets such as ownership of consumer items, types of dwelling, source of drinking water, and availability of electricity into a single index. It is recategorized as poorer and poor as lower, the middle as a middle, and richer and rich as higher. The age of the children were categorized as 0–11 months, 12–23 months, and 24–59 months. The birth order of the child is categorized as 1 (first), 2-3, 4–6, and ≥7.

Dependent variable was constructed as a dichotomous outcome; caretakers who sought care for their children with fever/ARI/diarrhea from formal health care (government health facilities, private hospitals/clinics, and nongovernmental organization clinics) were coded as one and those who did not seek formal care (including purchasing medicines from pharmacy, shop, and traditional healers) were coded as zero.

The DHS survey had ethical clearance from the National Research Ethics Review Committee. Ethical clearance for this study was obtained from the Institutional Review Board of Hawassa University. Official permission was also obtained from MEASURE/DHS to use the data for this analysis.

## 3. Result

Totally 11,645 women in reproductive age group (15–49 years) with their children under the age of five were included in the analysis, of which a total of 2,842 caregivers for children under the age of five years reported that their index child had one or more of the three common childhood illnesses in the two weeks preceding the survey. Majority, 9,668 (83%), of caretakers of the children were living in rural area. Seventy-six percent of the ages of the caretakers of the children were below 35 years of age. Caretakers of 70% of the children had no education and 25% had primary education level.

**Figure 1:**
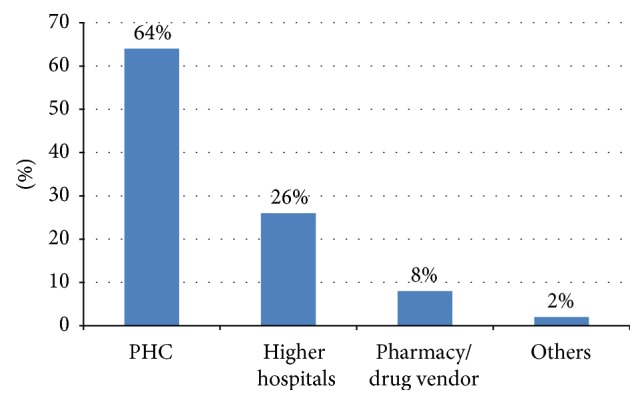
Among those who sought care, first option place of caretakers for the treatment of childhood illness. *N* = 1,226.

**Figure 2:**
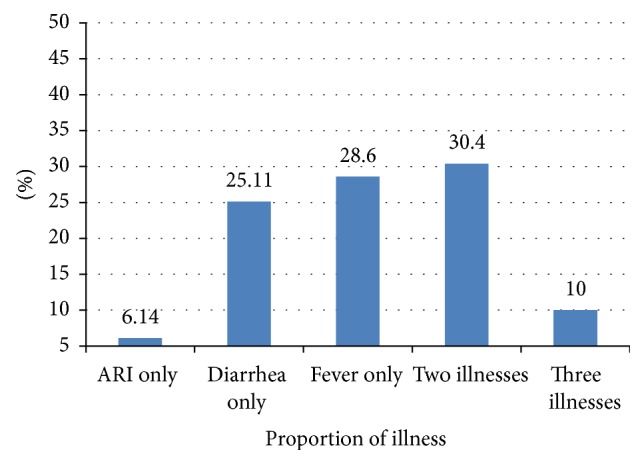
Proportion of children reported suffering from common childhood illness in the two weeks preceding the survey. *N* = 2,842.

Of 2,842 caregivers studied, 849 (29.87%; 95% CI: 28, 32%) sought health care for their ill children. Among those who sought care, the most frequent first option for treatment of a child with common childhood illness was primary health care facilities followed by higher hospital. For more details, please see Figure 1. Separate analysis for each illness showed that 1,416 (68.01%) children with fever, 1,044 (64.44%) with diarrhea, and 644 (83.41%) with ARI did not receive any modern treatment. As reported in Figure 2 there may be children with more than one illness; the total number of children for the separate analysis is not the same as the number of children with at least one common childhood illnesses.

As depicted in Table 1 there was a difference in seeking care by number of under-five children; those caretakers who had one under-five child seek health care more than those caretakers who had two and more under-five children. In <2 U5 children 726 (30.74%) and in ≥2 U5 children 123 (25.62%) sought formal care. This difference was statistically significant (Chi^2^ (1) = 5, *p* < 0.001).

In multivariable analysis, exposure to mass media, birth order of the child, and wealth were the statistically significant factors. Those caretakers who had exposure to mass media, for example, listening to the radio at least once a week, were more likely to seek formal care for their child with common childhood illness (AOR = 1.5; 95% CI: 1, 2.1). The higher the birth order of the child the lower the health care seeking behavior of caretakers for their children with common childhood illness (AOR = 0.5; 95% CI: 0.3, 1). Caregivers at a higher wealth index category are more likely to seek formal care for their child with common childhood illness (AOR = 1.6; 95% CI: 1.1, 2.4). For more details please see Table 2.

## 4. Discussion

Overall, less than one-third of caretakers of children under the age of five years with common childhood illness seek formal health care. Health care seeking from formal health care system was better for those caretakers who were exposed to mass media and with higher wealth status. Children born from large family size are also less likely to get immediate care from formal health facilities.

Appropriate health seeking behavior of caretakers is very low in this study compared to similar studies done in Nepal, Myanmar, Cambodia, Yemen, Tanzania, and Kenya [4, 17–21]. This might be because in Ethiopia 85% of the population is living in rural areas; the descriptive analysis of this study showed that (Table 1) majority of rural caretakers do not seek health care; that could be due to lack of awareness. Low literacy rate among caretakers who live in rural parts of Ethiopia was attributed to lack of awareness and eventually leads to low health seeking behavior. Moreover economic challenges and problem of accessing the health facilities are some of the reasons. This is because of physical and economical access to the health service is courage for the caretakers to seek care.

The study revealed that caretakers who had two and more under-five children are less likely to seek formal health care compared to those who had one. Formal health care seeking among caretakers are also associated with family size. The larger the size of the family, the lower the caretakers to seek formal health care. This shows that children born to large family size are less likely to get formal health care. This finding is in line with studies done in Tanzania [22]. This might be due to the fact that in Ethiopia women are responsible for taking care of their family and this is the routine activity done by mothers. As a result taking care of large family might lead to high work load and not giving much more attention for the sick child. The other possible reason might be financial constraint on visiting health facilities.

The result also revealed that those caretakers from higher wealth seek more formal health care compared to the lower wealth. This is also similar to the studies done in Nepal, Cambodia, and Ghana [4, 17, 23]. This is mainly because caretakers need resources to get service from health facilities.

Primary health care facilities were among the most common care provider. This result is also in line with different studies done in Cambodia, Ghana, and Tanzania [4, 16, 23]. This is because, in Ethiopia, government facilities are the major health care providers and PHC is relatively cheap in cost and reachable for the caretakers.

This study revealed that those caretakers who had exposure to mass media (listening to radio at least once a week) seek more formal care than those who do not. The finding is also similar to the study done in Cambodia [4]. Those caretakers, at least who have exposure to media, have better awareness of the importance of seeking formal care.

The limitations of this study include the data set lacks variable such as at what time caretakers sought medical advice. It is important to check how fast a caretaker acting for the illness. However, the rest of the variables are robust and representative of the country; as a result the findings of this study are generalizable.

In conclusion, health care seeking behavior of caretakers in Ethiopia is low; less than one-third of caretakers sough formal heath care. Among those who sought formal health care, primary health care facilities were the most common care providers. Exposure to mass media appears to have played a role in motivating health care seeking behavior and family planning is also important to maximize health seeking behavior of the caretakers.

## Figures and Tables

**Table 1:** Proportion of children under the age of five with common childhood illness who sought formal medical care by selected characteristics descriptive statistics with Pearson's Chi square.

Name of the variables	Sought formal care	Did not seek formal care	Pearson's Chi square
Age of the child			
0–11	187 (27.79)	486 (72.21)	Chi^2^ (2) = **3.2**
12–23	231 (32.17)	487 (67.83)	
24–59	423 (29.83)	995 (70.17)	
Sex			
Male	448 (30.46)	1,023 (69.54)	Chi^2^ (1) = **.49**
Female	401 (29.25)	970 (70.75)	
Number of U5 children			
≤2	726 (30.74)	1,636 (69.26)	Chi^2^ (1) = 4.97^*∗*^
>2	123 (25.62)	357 (74.38)	
Birth order of the child			
First	210 (41.67)	294 (58.33)	Chi^2^ (3) = 57.74^*∗*^
2-3	282 (31)	627 (69)	
4–6	255 (27.39)	676 (72.61)	
≥7	102 (20.48)	396 (79.52)	
Residence			
Urban	223 (50.23)	221 (49.77)	Chi^2^ (1) = 104^*∗*^
Rural	626 (26.11)	1,772 (73.89)	
Distance			
Not big problem	279 (39)	436 (61)	Chi^2^ (1) = 38^*∗*^
A big problem	570 (26.82)	1,555 (73.18)	
Transport			
Not a problem	244 (39.61)	372 (60.39)	Chi^2^ (1) = 35.51^*∗*^
Big problem	605 (27.19)	1,620 (72.81)	
Wealth			
Lower	322 (22.41)	1,115 (77.59)	Chi^2^ (2) = 100.32^*∗*^
Middle	135 (29.16)	328 (70.84)	
Higher	392 (43.5)	550 (58.39)	
Money			
Not a problem	285 (35.80)	511 (64.2)	Chi^2^ (1) = 18.49^*∗*^
Big problem	564 (27.58)	1,481 (72.42)	

^*∗*^Significant, *p* < 0.001.

**Table 2:** Multivariable analysis of factors associated with health seeking behavior of caretakers for their child with common childhood illness in children under the age of five in Ethiopia (from EDHS data 2011).

	Sought formal care	Did not seek formal care	COR (95% CI)	AOR (95% CI)
Age of the child in months				
0, 11	187 (27.79)	486 (72.21)	1	1
12, 23	231 (32.17)	487 (67.83)	1.3 (0.9, 1.8)	1.3 (0.9, 1.9)
24, 59	423 (29.83)	995 (70.17)	1.2 (0.8, 1.6)	1.1 (0.8, 1.6)
Sex of the child				
Male	448 (30.46)	1,023 (69.54)	1	1
Female	401 (29.25)	970 (70.75)	0.9 (0.7, 1.2)	0.9 (0.7, 1.2)
Birth order of the child				
First	210 (41.67)	294 (58.33)	1	1
2, 3	282 (31.02)	627 (68.98)	0.7 (0.5, 1.1)	0.8 (0.6, 1.3)
4, 6	255 (27.39)	676 (72.61)	0.7 (0.5, 1)^*∗*^	0.9 (0.6, 1.4)
≥7	102 (20.48)	396 (79.52)	0.3 (0.2, 0.5)^*∗*^	0.5 (0.3, 1)^*∗*^
Residence				
Urban	223 (50.23)	221 (49.77)	1	1
Rural	626 (26.11)	1772 (73.89)	0.4 (0.3, 0.7)^*∗*^	0.8 (0.4, 1.3)
Maternal education				
No education	497 (25.49)	1,453 (74.51)	1	1
Primary	274 (35.96)	488 (64.04)	1.5 (1.1, 2)^*∗*^	1.1 (0.8, 1.5)
Secondary and higher	78 (60)	52 (40)	4.2 (2.4, 7.3)^*∗*^	1.6 (0.6, 4)
Paternal education				
Not educated	328 (23.53)	1,066 (76.47)	1	1
Primary	347 (32.16)	732 (67.84)	1.3 (0.9, 1.7)	0.9 (0.7, 1.3)
Secondary	89 (48.63)	94 (51.37)	2.5 (1.4, 4.4)^*∗*^	1.3 (0.7, 2.5)
Higher	62 (50.41)	61 (49.59)	3.3 (1.8, 6)^*∗*^	1.4 (0.6, 3.1)
Wealth				
Lower	322 (22.41)	1,115 (77.59)	1	1
Middle	135 (29.16)	328 (70.84)	1.5 (1.1, 2.2)	1.5 (1, 2.1)^*∗*^
Higher	392 (41.61)	550 (58.39)	2.2 (1.6, 3)^*∗*^	1.6 (1.1, 2.4)^*∗*^
Maternal age				
15, 24	234 (34.21)	450 (65.79)	1	1
25, 29	285 (31.67)	615 (68.33)	0.8 (0.6, 1.2)	0.9 (0.6, 1.3)
30, 34	176 (30.34)	404 (69.66)	0.8 (0.6, 1.2)	1.1 (0.6, 1.7)
35, 49	154 (22.71)	524 (77.29)	0.5 (04, 0.7)^*∗*^	0.8 (0.5, 1.3)
Transport				
Not a problem	244 (39.61)	372 (60.39)	1	1
Big problem	605 (27.19)	1,620 (72.81)	0.7 (0.5, 0.9)^*∗*^	1 (0.7, 1.4)
Watching TV				
Not at all	544 (26.81)	1,485 (73.19)	1	1
Less than once a week	166 (30.97)	370 (69)	1.5 (1.1, 2.2)^*∗*^	1.2 (0.9, 1.8)
At least once a week	137 (49.82)	138 (50.18)	2.1 (1.3, 3.2)^*∗*^	1 (0.6, 1.7)
Listening to the radio				
Not at all	431 (27)	1,166 (73)	1	1
Less than once a week	234 (30)	547 (70)	1.1 (0.8, 1.5)	0.9 (0.7, 1.2)
At least once a week	183 (39.52)	280 (60.48)	1.9 (1.3, 2.7)^*∗*^	1.5 (1, 2.1)^*∗*^
Reading news				
Not at all	740 (28.2)	1,884 (71.8)	1	1
Less than once a week	84 (49.41)	86 (50.59)	2.7 (1.8, 4.3)^*∗*^	1.5 (0.9, 2.7)
At least once a week	24 (52.17)	22 (47.83)	1.8 (0.8, 4.2)	0.4 (0.1, 1.3)

^*∗*^Significant, *p* < 0.001.
